# Elevational Pattern of Leaf Mine Diversity on *Quercus variabilis* Blume at Baotianman, Henan, China

**DOI:** 10.3390/insects14010007

**Published:** 2022-12-21

**Authors:** Xiaona Chen, Miao Zhong, Lixing Cui, Jiasheng Xu, Xiaohua Dai, Xiaojing Liu

**Affiliations:** 1 Leafminer Group, School of Life Sciences, Gannan Normal University, Ganzhou 341000, China; 2 National Navel Orange Engineering Research Center, Ganzhou 341000, China; 3 Ganzhou Key Laboratory of Nanling Insect Biology, Ganzhou 341000, China; 4 Baotianman National Nature Reserve Administrative Bureau, Nanyang 474350, China

**Keywords:** leaf-mining insects, elevational pattern, species diversity, functional diversity, phylogenetic diversity

## Abstract

**Simple Summary:**

The patterns and causes of biodiversity variations along environmental gradients are hot topics for ecologists and biogeographers. Leaf miners are the specific insect guild that feed and live inside plant leaves. Although altitudinal diversity trends have been studied for many insect groups, few scientists have focused on the elevational diversity pattern of leaf-mining insects. To the best of our knowledge, there are no reports on the elevational distribution of leaf miners in China. Moreover, all previous work on the elevational change of leaf miners only focused on their abundance or species richness, without further analyses on their diversity indices, especially on phylogenetic diversity and functional diversity. The diversity of the leaf-mining insects on one dominant oak species in Central China was thus investigated through Hill numbers. The oak species hosted rich leaf miner species, and different leaf miners showed different elevational preferences. Most diversity metrics of leaf miners generally followed hump-shaped mid-peak elevational patterns.

**Abstract:**

The species composition and diversity pattern of leaf miners on dominant trees in China are poorly understood. Using Hill-based diversity metrics, the elevational patterns of taxonomic, phylogenetic, and functional diversity for leaf miners on *Quercus variabilis* Blume at Baotianman were systematically analyzed. Leaf mine types belonged to ten genera and seven families. Different leaf miners had different elevational preferences. Most taxonomic and phylogenetic Hill diversity indices had typical hump-shaped elevational patterns, with a peak at the middle elevation of approximately 875 m. No functional Hill diversity indices presented significant linear or nonlinear trends with altitude. The driving factors behind the elevational distribution patterns of leaf miners require further work.

## 1. Introduction

The patterns and causes of biodiversity variations along environmental gradients are hot topics in ecology and biogeography [1,2,3,4]. The elevation gradient includes the gradient effects of various environmental factors, such as temperature, moisture, and light [5]. Therefore, studying elevational biodiversity change is essential for exploring the influencing factors behind biodiversity-environmental gradient relationships [3,6]. For host-specific herbivorous insects, their elevational distribution is also affected by their host plants’ elevational distribution [7,8,9,10]. The diversity of most herbivorous insects shows the following elevational patterns: (1) diversity peaks at lower elevations; (2) diversity peaks at middle elevations; (3) diversity peaks at higher elevations; and (4) diversity has no apparent relationship with elevation [10,11,12,13,14]. In the current research on the elevational patterns of herbivorous insects, exophagous insects are relatively well studied, while endophagous insect research is mainly concentrated on wood-boring insects and gall-forming insects [7,15,16].

Leaf-mining insects are another important endophagous group. They feed and live inside host plant leaves during either young or whole larval stages [17,18,19,20,21]. More than 10,000 leaf-mining species are found in approximately 50 families belonging to the four largest orders of Insecta: Lepidoptera, Coleoptera, Diptera, and Hymenoptera [21]. However, the number of leaf-mining species is far higher than what is currently known [21]. The feeding trails of leaf miners are known as leaf mines [21]. The mine characteristics are usually family- or genus-specific [22], which are valuable clues for insect identification. The mines can remain on the leaves for a long time, and some mine traces are kept in fossil leaves. According to leaf mines, both ecologists and paleontologists can reconstruct the feeding habits, life history, and interspecific relationships of leaf-mining insects [23,24,25,26,27,28]. Moreover, most leaf-mining insects are monophagous or oligophagous, with high host specificity [28,29]. Therefore, leaf-mining insects can be used as model organisms to study species interactions, coevolution, and environmental adaptation [30,31,32,33].

There are fewer studies focusing on the elevational distribution of leaf-mining insects, most of which are on the population density of a single species, usually an important pest [34,35,36]. Diversity at the community level is rarely analyzed for leaf-mining insects at different elevations [37,38]. Leaf-mining insects are sensitive to environmental changes, and how elevation affects the distribution of leaf-mining insect communities is poorly understood [37,38]. Elevational variation in climatic factors [34,35,36,37,38,39,40,41,42,43], plant characteristics [37,38,41,42,43,44,45,46], natural enemies [36,42,43], and human activities [35], may contribute differently to the elevational distribution of leaf miners. Furthermore, some factors have been implicated as the underlying causes of elevational diversity [12], such as source-sink dynamics [47] and evolutionary history [48], which may also affect the distribution of leaf-mining insects.

Fagaceae plants are widely distributed, consisting of 900–1000 species in 7–12 genera [49]. Many Fagaceae plants are the dominant species in forest communities in the Northern Hemisphere, covering tropical, subtropical, and temperate climate zones [50]. According to the ‘plant apparency hypothesis’, such dominant plants are susceptible targets to leaf-mining insects [51,52,53]. For instance, *Quercus robur* hosts 36 leaf-mining species in the UK [54]; *Q. crispula* hosts over 12 leaf-mining species and *Q. dentata* hosts 9 species in Japan [55,56,57]; *Q. falcata* hosts 18 species, *Q. nigra* hosts 17 species and *Q. hemisphaerica* hosts 15 species in northern Florida; and oaks host 15 genera and nine families of leaf miners, while a single oak species can have 2 to 18 leaf miners in California [58]. To date, more than 400 leaf-mining insects have been globally discovered on Fagaceae plants, and they cover nearly all the typical leaf-mining families (Xiaohua Dai et al., unpublished data).

Chinese cork oak (*Q. variabilis*) is one of the most extensively distributed deciduous broad-leaved tree species in East Asia [59,60]. Its distribution latitude is from 22° to 42° N, and its continuous distribution range is from 24.43° to 40.25° N, covering the southern subtropical, central subtropical, northern subtropical, warm temperate, and medium temperate zones [61,62]. The vertical distribution of *Q. variabilis* is from 50 m to 3000 m [60]. Similar to other Fagaceae plants, leaf-mining insects are diverse on *Q. variabilis* [63].

To the best of our knowledge, there are no reports on the elevational distribution of leaf miners in China. Moreover, all previous work on the elevational change of leaf miners only involved their abundance or species richness, without further analyses on their diversity indices, especially on phylogenetic diversity and functional diversity. In our study, we used *Q. variabilis* as an example to explore the relationships between elevation and the following: (i) the abundance of different leaf miners; (ii) the taxonomic diversity of leaf miners; (iii) the phylogenetic diversity of leaf miners; and (iv) the functional diversity of leaf miners. We intended to answer the following questions: (i) Are the elevational patterns of leaf-mining insects the same as those of other herbivorous insects? (ii) Do their elevational distributions fit the “mid domain model”, “monotonic decreasing model”, or “monotonic increasing model”? (iii) Do different diversity metrics respond differently to elevation?

## 2. Materials and Methods

### 2.1. Study Site

**Figure 1:**
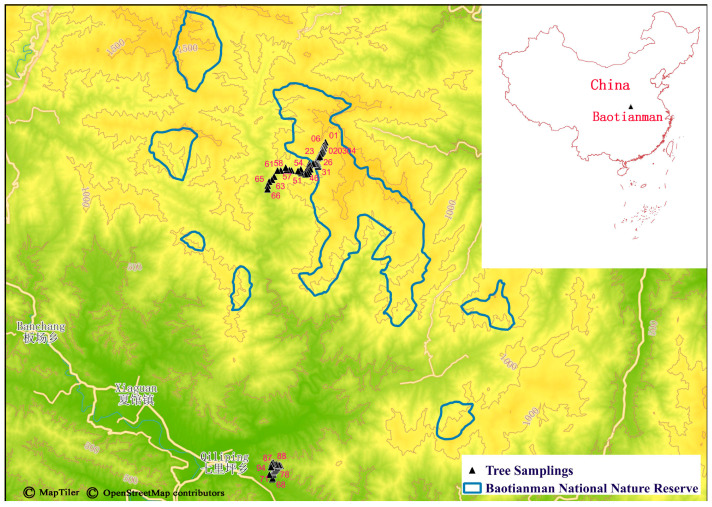
Sampling areas of leaf mines on *Quercus variabilis* Blume at Baotianman, Henan. The numbers in pink indicate the order of tree samplings. The reserve boundary was provided by Baotianman National Nature Reserve Administrative Bureau. The map was constructed using QGIS 3.26.3 [74]. QGIS MapTiler Plugin can obtain OpenStreetMap data from the OpenMapTiles project (openstreetmap.org). The base maps, terrain, and contours for this plugin are available from the MapTiler Cloud under the Open Database License.

Baotianman (33°20′~33°36′ N, 111°47′~112°04′ E, 500~1845 m a.s.l) is located in Neixiang County in southwestern Henan Province, Central China (Figure 1). It is at the southern slope of the Funiu Mountains in the eastern extension of the Qinling Mountains (the natural boundary between South and North China) [64]. It has a continental monsoon climate with four distinct seasons [64], with an average annual temperature of 15.1 °C, an average annual precipitation of approximately 900 mm, an average annual evaporation of 991.6 mm, and an average annual relative humidity of 68% [65,66,67,68]. The climate is between the northern subtropical and warm temperate zones [64,69]. The corresponding vegetation transitions from deciduous broadleaf forest to evergreen broad-leaved forest [70]. Baotianman National Nature Reserve has rich species, with 223 families, 1002 genera, 2771 species of plants, and over 1700 species of animals, including 1500 species of insects [71]. *Q. variabilis* is one of the primary constructive and dominant species in Baotianman and the surrounding areas [64,67,68,72,73].

### 2.2. Sampling of Leaves with Mines

In September 2020, according to the known distribution areas of *Q. variabilis* and the map information (Figure 1), we set up two transects from 300 to 1350 m a.s.l at Baotianman, i.e., Baotianman Scenic Area (600 m–1350 m) and Houyemiao, Qiliping County (300 m–600 m). Part of the first transect was inside the experimental zone of Baotianman National Nature Reserve. The two transects form a continuous elevation gradient (Figure S1). First, we tried to find some flat plots at certain altitudes to perform the investigation. However, the slopes are very steep in the Baotianman Scenic Area, and the highway is meandering. *Q. variabilis* trees are scattered along the highway, especially in regions of human disturbance. Therefore, we walked on the highway from the mountain top to the mountain foot to sample leaves with mines present from nearly all accessible *Q. variabilis* trees. The distances between two neighboring sampled trees were relatively even (mean ± SD: 111 ± 111 m, with a range of 0–561 m, SD: mean = 1 indicating an even distribution), although we did not plan it that way. There are almost no *Q. variabilis* trees along the highway in the valley area due to high human disturbance. Therefore, we have to set up another low-altitude transect at Houyemiao. At the Houyemiao transect, *Q. variabilis* trees are also scattered except on the mountain top. Therefore, we also tried to sample the trees evenly along the mountain road at Houyemiao (distance between two sampled neighbors, mean ± SD: 79 ± 64 m, with a range of 9–221 m, SD: mean = 0.81 indicating a nearly even distribution). We carried out continuous sampling along the whole elevational range from 300 m to 1350 m.

Most sampled *Q. variabilis* trees were saplings (tree height between 2 and 3 m), so we collected all leaves with leaf mines from the whole saplings. For some adult trees (tree height > 3 m), we collected all mined leaves from only accessible branches (branch height to the ground < 3 m, analogous to one tree sapling) without using additional long-reach pruning tools. The sampled trees were labeled consecutively as 01, 02, 03… 92, 93, 94 (Table S1). Occasionally, there were two or three trees located in the same place, and we only kept one tree with the richest leaf mines. However, we also analyzed the mining diversity pattern with or without the removal of tree samplings at the same location, and the results were almost the same. The location and elevation of each tree sample was measured and recorded with the Aowei Interactive Map app and a Garmin GPS device. Leaf samples with leaf mines were placed in plastic self-sealable bags, and each bag was blown with the appropriate gas to ensure the freshness of the sampled leaves.

### 2.3. Leaf Mine Classification

Due to long-term coevolution and interspecific differentiation, leaf miners have formed various mine forms, reflected in the feeding parts and mine shape [17,18,24,75]. For example, different leaf-mining larvae may feed on different vertical leaf parts and make leaf mines of various depths [18,76], including upper-surface mines in the palisade mesophyll [77], lower-surface mines in the sponge mesophyll [78], epidermal mines in the epidermis [79], and full-depth mines consuming both palisade and spongy mesophyll tissues [21]. Leaf mine shapes can be divided into three main categories: linear mines, blotch mines, and linear blotch mines [18]. Linear mines are caused by the consistent one-directional feeding of leaf-mining larvae; blotch mines are made when larvae feed in multiple directions; and linear blotch mines are transitional types between linear mines and blotch mines [18].

Based on the above and other leaf mine characteristics (Table S2) of known insect inducers from publications, websites, and our previous rearing records, the corresponding leaf-mining insect groups in our collections were preliminarily identified. Then, we consulted leaf miner experts (see Acknowledgments for names and institutes) to verify the identification. By far, we could only identify the leaf mine to the genus level. Ten leaf mine types and their related genera of leaf-mining insects on *Q. variabilis* at Baotianman were then obtained: LM01 (*Acrocercops*), LM03 (*Caloptilia*), LM05 (*Dactylispa*), LM06 (*Ectoedemia*), LM08 (*Phyllonorycter*), LM10 (*Stigmella*), LM11 (*Tischeria*), LM12 (*Trachys*), LM15 (*Chrysopeleia*), and LM19 (*Profenusa*) (Table S3). Sample leaves with the same leaf mine type were scanned together with an EPSON 10000XL, and the scanned images were saved.

### 2.4. Leaf Mine Area Measurement

Twenty leaves with one complete mine image were selected for each leaf mine type, and the mining part was filled in red (or a color that differed markedly from the healthy part of the leaf), while the other leaf part was filled in green with Adobe Photoshop 2021 (CS5.1). The processed pictures were then imported into WinFolia 2016b Pro (Regent Instruments Canada Inc., Quebec City, QC, Canada). Setting green as the healthy color, red as the mine color, and white as the background color, the area of the mining part for each mine type could be calculated from the leaf area and health rate: leaf mine area = leaf area ∗ (1 − health rate). The area of each leaf mine type was the average value of twenty leaves (Table S2). For the leaf mine type without twenty leaf samples in this investigation, we use additional *Q. variabilis* leaf samples with the corresponding mine type in other years. 

### 2.5. Data Analysis

#### 2.5.1. Taxonomic Hill Diversity of Leaf Mines

Based on the number of each leaf mine type on each *Q. variabilis* tree (Table S1), the following taxonomic Hill diversity indices of leaf mines were calculated for each tree individual with the “hillR” R package [80]. The taxonomic Hill numbers through different orders (*q* values) have different meanings: (1) *q* = 0, species richness and it reflects the diversity of all species; (2) *q* = 1, Shannon entropy index and it reflects the diversity of common species; and (3) *q* = 2, inverse Simpson’ dominant index and it reflects the diversity of dominant species [80,81,82].

#### 2.5.2. Phylogenetic Hill Diversity of Leaf Mines

The taxonomic status (~Order/Superfamily/Family/Genus) of the leaf-mining insect groups on each leaf mine type (Table S3) was used to obtain the phylogenetic tree (Figure S2) using the ‘as.phylo.formula’ function of the “ape” R package [83]. The following phylogenetic Hill numbers of leaf mines were then computed for each tree individual by the “hillR” R package [80]. The phylogenetic Hill numbers through different orders (*q* values) are also closely related to different phylogenetic diversity indices: (1) *q* = 0, the phylogenetic Hill number is related to Faith’s phylogenetic diversity; (2) *q* = 1, the phylogenetic Hill number is related to Allen’s phylogenetic entropy; and (3) *q* = 2, the phylogenetic Hill number is related to Rao’s quadratic entropy [82,84,85,86].

#### 2.5.3. Functional Hill Diversity of Leaf Mines

Based on the functional characteristics of each leaf mine type (Table S2), the trait-clustering dendrogram among different leaf mine types was constructed by “FD” R package [87,88] (Figure S3). The following functional Hill numbers through different orders (*q* values) of leaf mines were then calculated for each tree individual by the “hillR” R package [80]: (1) *q* = 0, *FAD* (functional attribute diversity); (2) *q* = 1, the related functional diversity index is still unclear; and (3) *q* = 2, the function Hill number is related to Rao’s quadratic entropy and weighted Gini–Simpson’s index [82,89,90,91]

#### 2.5.4. Elevational Diversity Pattern

A piecewise model is a regression model used to clarify whether the relationship between one or more explanatory variables is piecewise linear [92]. The value corresponding to the turning point in the piecewise fitting process is the break point [93]. The “segmented” R package provides tools for estimating and summarizing generalized linear models with piecewise relationships, and there are no restrictions on the number of segmented variables and the number of change points [93]. Regression splines are used to estimate break points (knots in spline terminology) when the sample point interval is known [93], and splines with a single break point can be useful statistical tools for modeling linear predictors in generalized linear models [94]. In addition, there are four other ways to evaluate break points [92]. The piecewise model is especially appropriate for our consecutive samplings along the elevational gradient.

The relationship between each Hill diversity index and the corresponding elevation of each sampled tree was fitted with the piecewise linear model. When the piecewise relationship was not significant (*p* > 0.1), the simple linear model was alternatively used for the fitting. All fittings were run with the “segmented” R package [92,93,95,96]. In some cases, nonlinear regressions might be more appropriate to fit the above relationships. The nonlinear fitting results with a number of models, such as linear, quadratic, power, exponential, Von Bertalanffy, Michaelis–Menten, logistic, Gompertz, Gaussian, and Hill, were quickly checked with the Past software [97] and appropriate models were selected according to the curves, *AIC* values and *R*-squared values.

#### 2.5.5. Spatial Autocorrelation Analysis

The presence of spatial autocorrelation in geospatial data might make non-significant regression relationships show false significant results [98,99,100,101]. Therefore, potential spatial autocorrelation in the elevational distribution of leaf mine diversity indices should be considered. The latitude and longitude of each sample tree were converted to the planar XY position (unit: m) using the “PBSmapping” R package [102]. Based on the planar coordinates of each tree, the ‘modifiedttest’ function in the “SpatialPack” R package [103] was used to assess the spatial autocorrelation between taxonomic, phylogenetic, or functional Hill numbers and elevation. The modified *t* test corrects the Pearson’s correlation for spatial autocorrelation based on Dutilleul’s method [104,105]. The ‘modifiedttest’ function also provides Moran’s index for both spatial variables [103].

#### 2.5.6. Community Similarity Analysis

The community similarity index of leaf mine types and elevation differences between each tree pair (*j*, *k*) were calculated and output as matrices using Past 4.11 [97]. Community similarity was measured as the Bray-Curtis index (*d_jk_*) as follows: $d_{jk}=1-\frac{\sum_{i}^{\;}\left|x_{ji}-x_{ki}\right|}{\sum_{i}^{\;}\left(x_{ji}+x_{ki}\right)}$ where *x_mn_* is the abundance of leaf mine type *n* on tree *m*. The lower triangular portion of each matrix was extracted using the “gdata” R package [106]. The relationship between the community similarity index and elevation differences was then fitted with the simple linear model.

The above taxonomic diversity, phylogenetic diversity, and functional diversity analyses, model fitting and community similarity analyses were carried out with R 4.2.1 [107] in the graphic interface of RStudio [108].

## 3. Results

### 3.1. Number of Leaf Mine Types at Different Transects

In total, there were 3713 leaf mines and ten leaf mine types (i.e., ten leaf-mining genera) on 89 individuals of *Q. variabilis* at Baotianman along an elevational gradient from 300 m to 1350 m (Table 1, Tables S4 and S5). The number of leaf mine types per tree was mean ± SD: 4.6 ± 1.2. The maximum number of leaf mine types per tree was seven, while the minimum number of leaf mine types per tree was one. *Phyllonorycter* was present in nearly all sampled trees (88/89). In the Baotianman Scenic Area (600–1350 m a.s.l), there were a total of nine leaf mine types, without *Dactylispa*. The number of leaf mine types per tree was mean ± SD: 4.5 ± 1.1. At Houyemiao, Qiliping County (300–600 m a.s.l), there were a total of nine leaf mine types, without *Caloptilia*. The number of leaf mine types per tree was mean ± SD: 4.6 ± 1.4. There were no significant differences between the mean number of leaf mine types per tree among the two transects (*t* = 0.773, *p* = 0.442 > 0.05).

### 3.2. Elevational Pattern of Mine Numbers

**Figure 2:**
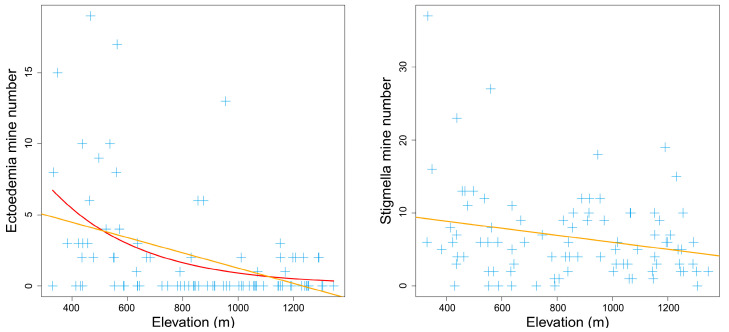
Elevation decreasing pattern of mine numbers on *Quercus variabilis* Blume. The orange line was fitted with the piecewise linear model, while the red line was fitted with the nonlinear quadratic model.

**Figure 3:**
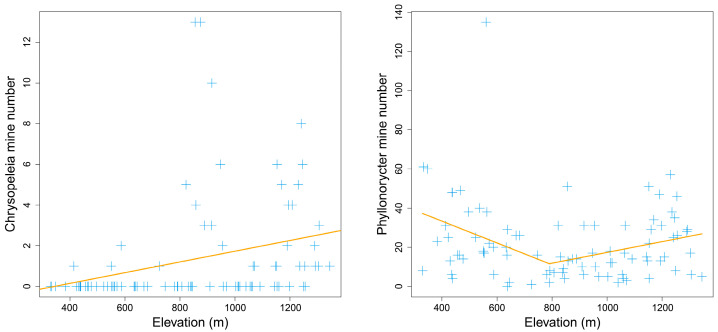
Elevation increasing and V-shaped pattern of mine numbers on *Quercus variabilis* Blume. The orange line was fitted with the piecewise linear model.

Within the elevational range from 300 m to 1350 m, the total number of leaf mines on *Q. variabilis* had no apparent elevational pattern (*p* > 0.1). However, the individual numbers of different leaf mine types presented different elevational patterns. The numbers of *Ectoedemia* and *Stigmella* mines significantly decreased with elevation (piecewise model: *P*_Ectoedemia_ = 0.021 *<* 0.05, *P*_Stigmella_ = 0.057 < 0.1) (Figure 2). For *Ectoedemia*, the piecewise model was better than the nonlinear quadratic model (*AIC_piecewise_*: 363 < *AIC_quadratic_*: 481). The number of *Chrysopeleia* mines significantly increased with elevation (*P*_Chrysopeleia_ = 0.015 *<* 0.05) (Figure 3). The number of *Phyllonorycter* mines had a minimum value at middle elevations of 786 m (*P*_Phyllonorycter_ = 0.029 *<* 0.05) (Figure 3). In contrast, the numbers of *Acrocercops*, *Tischeria*, and *Profenusa* mines had no apparent elevational patterns (*p* > 0.1). The rare leaf mine types *Caloptilia* and *Dactylispa* occasionally existed in high- and low-elevation areas, respectively, while *Trachys* was more common at low altitudes.

### 3.3. Elevational Pattern of Taxonomic Hill Diversity

There was no significant spatial autocorrelation between any taxonomic Hill numbers and elevation (*q* = 0: *F* = 0.0085, *DF* = 175.4, *p* = 0.927 > 0.1; *q* = 1: *F* = 0.0186, *DF* = 31.3, *p* = 0.893 > 0.1; *q* = 2: *F* = 0.0203, *DF* = 19.6, *p* = 0.888 > 0.1). Most Moran’s indices of each taxonomic Hill number were generally around 0, indicating unapparent spatial autocorrelation of taxonomic Hill diversity (Table S6).

**Figure 4:**
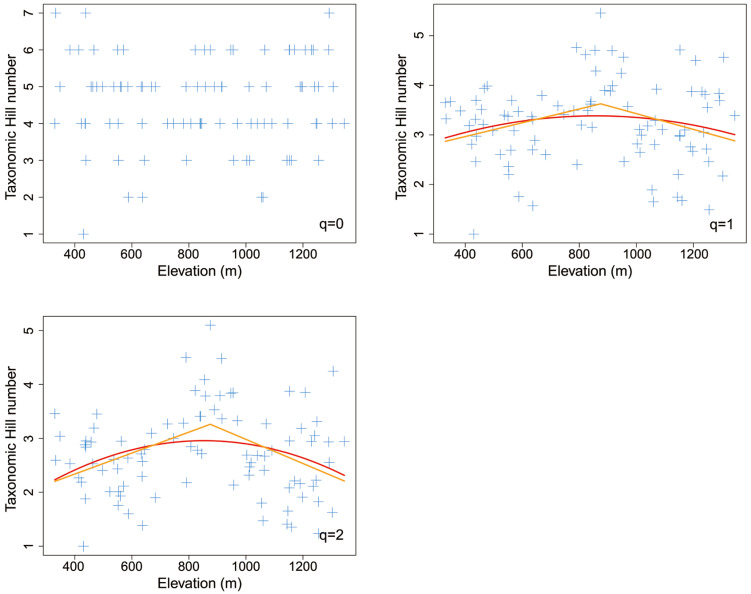
The elevational pattern of taxonomic Hill numbers for leaf mines on *Quercus variabilis* Blume. The orange line was fitted with the piecewise linear model, while the red line was fitted with the nonlinear quadratic model.

Both the piecewise model and the nonlinear model showed that most taxonomic Hill numbers peaked at the middle elevation of about 900 m (Figure 4). For Shannon entropy index (*q* = 1), the piecewise model was better than the nonlinear quadratic model (*AIC_piecewise_*: 224 < *AIC_quadratic_*: 227). For inverse Simpson’ dominant index (*q* = 2), the piecewise model was also better than the nonlinear quadratic model (*AIC_piecewise_*: 210 < *AIC_quadratic_*: 215).

Piecewise linear models indicated that both Shannon entropy index (*q* = 1) and inverse Simpson’ dominant index (*q* = 2) peaked at the middle elevation of 875 m, showing significant hump-shaped elevational patterns (*p* < 0.1) (Table 2). In contrast, taxonomic species richness (*q* = 0) did not show apparent elevational patterns (*p* > 0.1) (Figure 4).

### 3.4. Elevational Pattern of Phylogenetic Hill Diversity

There was no significant spatial autocorrelation between any phylogenetic Hill numbers and elevation (*q* = 0: *F* = 0.612, *DF* = 53.8, *p* = 0.438 > 0.1; *q* = 1: *F* = 0.268, *DF* = 14.0, *p* = 0.613 > 0.1; *q* = 2: *F* = 0.185, *DF* = 12.1, *p* = 0.675 > 0.1). Most Moran’s indices of each phylogenetic Hill number were generally around 0, indicating unapparent spatial autocorrelation of phylogenetic Hill diversity (Table S6).

**Figure 5:**
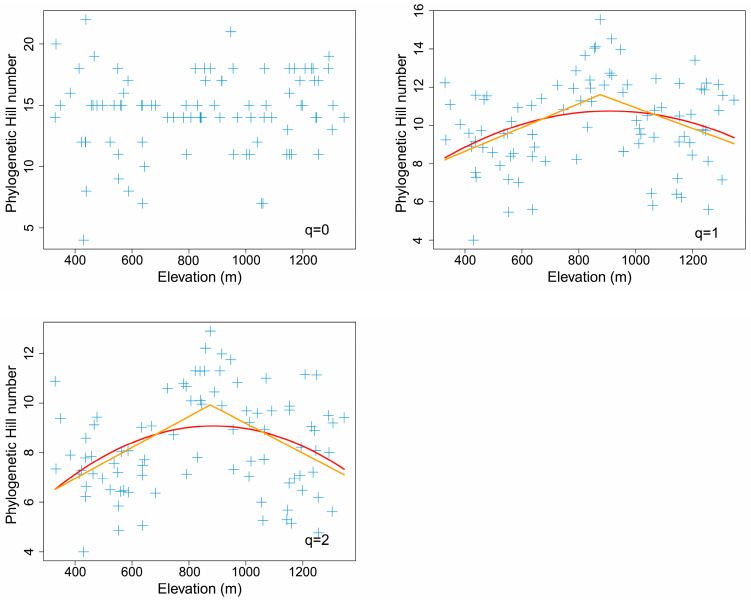
The elevational pattern of phylogenetic Hill numbers for leaf mines on *Quercus variabilis* Blume. The orange line was fitted with the piecewise linear model, while the red line was fitted with the nonlinear quadratic model.

Both the piecewise model and the nonlinear model showed that most phylogenetic Hill numbers reached a maximum at the middle elevation of about 900 m (Figure 5). For Allen’s phylogenetic entropy (*q* = 1), the piecewise model was better than the nonlinear quadratic model (*AIC_piecewise_*: 396 < *AIC_quadratic_*: 401). For Rao’s quadratic entropy (*q* = 2), the piecewise model was also better than the nonlinear quadratic model (*AIC_piecewise_*: 363 < *AIC_quadratic_*: 372).

Piecewise linear models indicated that both Allen’s phylogenetic entropy (*q* = 1) and Rao’s quadratic entropy (*q* = 2) peaked at the middle elevation of 875 m, showing significant hump-shaped elevational patterns (*p* < 0.1) (Table 2). In contrast, Faith’s phylogenetic diversity (*q* = 0) did not show apparent elevational patterns (*p* > 0.1) (Figure 5).

### 3.5. Elevational Pattern of Functional Hill Diversity

**Figure 6:**
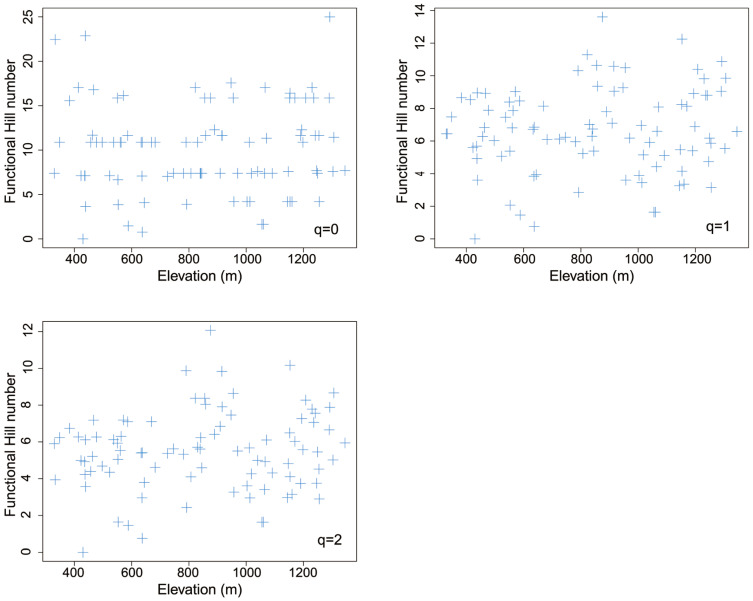
The elevational pattern of different total functional diversity indices for leaf mines on *Quercus variabilis* Blume.

There was no significant spatial autocorrelation between any functional Hill numbers and elevation (*q* = 0: *F* = 0.100, *DF* = 145.5, *p* = 0.752 > 0.1; *q* = 1: *F* = 0.493, *DF* = 40.4, *p* = 0.487 > 0.1; *q* = 2: *F* = 0.518, *DF* = 27.6, *p* = 0.478 > 0.1). Most Moran’s indices of each functional Hill number were generally around 0, indicating unapparent spatial autocorrelation of functional Hill diversity (Table S6). No functional Hill diversity indices showed significant linear or nonlinear trends with elevation (*p* > 0.1, Table 2, Figure 6).

### 3.6. Relationship between Leaf Mine Community Similarity and Elevation Difference

The similarity of leaf mine communities on *Q*. *variabilis* at Baotianman showed no apparent relationship with elevation difference (*p* > 0.1).

## 4. Discussion

Seven families and ten genera of leaf-mining insects were collected in this study (Table 1 and Table S3), including *Acrocercops* (Gracillariidae), *Caloptilia* (Gracillariidae), *Dactylispa* (Chrysomelidae), *Ectoedemia* (Nepticulidae), *Phyllonorycter* (Gracillariidae), *Stigmella* (Nepticulidae), *Tischeria* (Tischeriidae), *Trachys* (Buprestidae), *Stilbosis* (Cosmopterigidae), and *Profenusa* (Tenthredinidae), which have been discovered on *Q*. *variabilis* at Baotianman, supporting the hypothesis that dominant plants should have rich leaf miners [51].

The abundance and diversity along the elevational gradient varied for different animal groups. There were nine elevational diversity modes for birds, including increasing, decreasing, mid-peak, mid-valley, low-plateau, low-valley, high-plateau, high-valley, and nonsignificant modes [1,109]. Mid-peak is the dominant elevational diversity mode for nonflying small mammals [110,111]. Decreasing and mid-peak are the two main elevational diversity trends for bats [111]. Decreasing is the dominant elevational diversity trend for reptiles [111,112]. The elevational diversity patterns of geometrid moths could have decreased, mid-peak, and other complicated shapes [113]. Without regard to the specific leaf-mining species, the total number of leaf mines on *Q. variabilis* at Baotianman had no apparent elevational patterns, but the number of leaf mines on *Nothofagus pumilio* was negatively correlated with elevation [46]. With the increase in elevation difference, the similarity of the leaf miner community on *Q. variabilis* at Baotianman also showed no apparent pattern.

Different leaf-mining insect groups on *Q*. *variabilis* at Baotianman had different elevation preferences, and their abundance also reflected different altitudinal distribution patterns. The abundance of *Ectoedemia* and *Stigmella* had a peak at low elevations and decreased with increasing elevation. Similarly, the populations of *Leucopteru coffeellu* were larger at low elevations [36,42]. Both *Liriomyza sativae* and *L. trifolli* were more abundant in mid-low elevational domains [39]. The distributions of *Coptodisca lucifluella* and *Phytomyza ilicis* were also negatively correlated with elevation [34,43]. In contrast, the individual numbers of *L. huidobrensis*, *Caloptilia bryonoma*, *Lyonetia lechrioscia* and one unidentified leaf miner on *Doryphora sassafras* were predominant at high altitudes [37,39,40], such as *Chrysopeleia* in our study. The presence of *Tuta absoluta* and *Platynotocis angulipennis* showed no altitudinal trends [37,44], similar to *Acrocercops*, *Tischeria*, and *Profenusa* in our study. The abundance of *Phyllonorycter* was lowest at middle altitudes. Such a mid-valley pattern is not typical in leaf-mining insects.

Different leaf miners may have different ecological niches and perform differently under different environmental conditions. For example, some leaf-mining species only survive, develop, or reproduce in cooler places, while others adapt to warmer places, which may explain their differences in elevational distribution [34,38,40,42]. Precipitation at different altitudes may affect the population load of leaf miners [38,42,43]. Different climatic factors could also interact to change the altitudinal pattern of leaf miners [38]. The quantity and quality of plants and the pressure of natural enemies may alter the elevational pattern of leaf miners [38,42,43]. Climates can have indirect impacts on leaf miners through their effects on host plants and natural enemies at different elevations [38,42,43]. However, some leaf miners are highly adaptative and can expand their elevational range [39,44]. Human management could also alter leaf miners’ elevational distribution pattern [35,44]. In addition, the same leaf miner may show different shapes of elevational distribution curves in different places, especially when the elevational ranges are very different [36,42].

The taxonomic species richness of leaf-mining insects may decrease with altitude [38] or have no altitudinal trends [37,38]. In our study, the species richness (*q* = 0) of leaf miners on *Q*. *variabilis* did not vary significantly with elevation, but the Shannon entropy index (*q* = 1) and the inverse Simpson dominant index (*q* = 2) showed mid-peak distribution patterns. Such hump-shaped trends are common in invertebrates [114,115,116] and vertebrates [11,110,111,117,118].

The Faith’s phylogenetic diversity (*q* = 0) of leaf miners on *Q*. *variabilis* at Baotianman also did not vary significantly with elevation. However, all other phylogenetic Hill numbers (*q =* 1 and *q* = 2) of leaf mines peaked at middle elevations, similar to the results of some plants but different from many other animals. At the global scale, the Faith’s phylogenetic diversity of birds along elevation gradients is dominated by a low-plateau pattern, while the *MPD* (mean phylogenetic distance) of birds along elevation gradients is dominated by low-plateau pattern, mid-peak pattern, and high-valley pattern [109]. However, both *sesPD* (standardized effect size of Faith’s phylogenetic diversity) and *sesMPD* (standardized effect size of mean phylogenetic distance) of birds generally increased with increasing elevation [109]. In a temperate mountain forest in China, both the *sesMPD* and the Faith’s phylogenetic diversity of moths increased with increasing elevation [113]. 

None of the functional Hill numbers of leaf miners on *Q*. *variabilis* at Baotianman changed significantly with altitude. In contrast, the *FRic* (functional richness) and *FDis* (functional dispersion) of birds along elevational gradients were dominated by mid-peak and low-plateau patterns [109]. Both *sesFRic* (standardized effect size of functional richness) and *sesFDis* (standardized effect size of functional dispersion) of birds generally increased with increasing elevation [109].

Different components (taxonomic, phylogenetic or functional) of leaf mine diversity did not respond to the elevational gradient consistently. Such inconsistent patterns are also found in some previous biodiversity studies [119].

In conclusion, different leaf-mining insect groups might adapt to different elevational ranges, and the hump-shaped distribution pattern was typical in leaf-mine diversity along the elevational gradient. By far, we could identify the leaf mine to only the genus level. In the future, we will try to rear the leaf-mining insects and identify them with DNA barcodes. We can also further connect the distribution of leaf mine diversity with the variation in ecological factors and plant traits along the elevational gradient. Therefore, we can better understand the patterns and mechanisms of biodiversity.

## Figures and Tables

**Table 1:** Detailed information of the two transects for leaf mine sampling on *Quercus variabilis* at Baotianman.

Transect	Elevational Range (m)	Number of Sample Trees	Total Number of Leaf Mine Types	Mean Number of Leaf Mine Types per Tree (M)	Standard Deviation of M
Baotianman Scenic Area	600–1350	61	9	4.5	1.1
Houyemiao, Qiliping County	300–600	28	9	4.6	1.4
Total	300–1350	89	10	4.6	1.2

**Table 2:** Piecewise linear relationships between leaf mine diversity and elevation.

Diversity Category (*^q^D*)	*q* Value	*p* Value	Break Point (m)
	0	0.276	-
Taxonomic Hill numbers	1	0.072	875
	2	0.006	875
	0	0.326	-
Phylogenetic Hill numbers	1	0.002	875
	2	0.000	875
	0	0.119	-
Functional Hill numbers	1	0.910	-
	2	0.157	-

## Data Availability

The data supporting this study’s findings are available in the Supplementary Materials. The R codes are available on request from the corresponding author, X.D.

## Supplementary Materials

The following supporting information can be downloaded at: https://www.mdpi.com/article/10.3390/insects14010007/s1, Figure S1: Sampling trees of leaf mines on *Quercus variabilis* Blume at Baotianman, Henan. Figure S2: Phylogenetic tree of leaf-mining insects on *Quercus variabilis* Blume. Figure S3: Clustering dendrogram of leaf-mining insects on *Quercus variabilis* Blume based on functional traits. Table S1: Number of different mine types on *Quercus variabilis* Blume at different elevations. Table S2: Functional characteristics of different leaf mine types on *Quercus variabilis* Blume. Table S3: Taxonomic status of different leaf mine types on *Quercus variabilis* Blume. Table S4: Number of leaf miner genera on *Quercus variabilis* Blume at different altitudes in the Baotianman Scenic Area. Table S5: Number of leaf miner genera on *Quercus variabilis* Blume at different altitudes at Houyemiao, Qiliping County. Table S6 Moran’s index output from the ‘modifiedttest’ function in the “SpatialPack” R package.
