# Supplementary material for: Elevational Pattern of Leaf Mine Diversity on Quercus variabilis Blume at Baotianman, Henan, China
Source: Insects. 2022 Dec 21;14(1):7. doi: 10.3390/insects14010007 (PMC9861204; doi:10.3390/insects14010007)

Figure S1. Sampling trees of leaf mines on *Quercus variabilis* Blume at Baotianman, Henan (a-c: Baotianman Scenic Area; d: Houyemiao, Qiliping County). The numbers in pink indicated the order of tree samplings. The base map of contours was from MapTiler. The imagery was from Bing Virtual Earth. The map was constructed using QGIS 3.26.3.

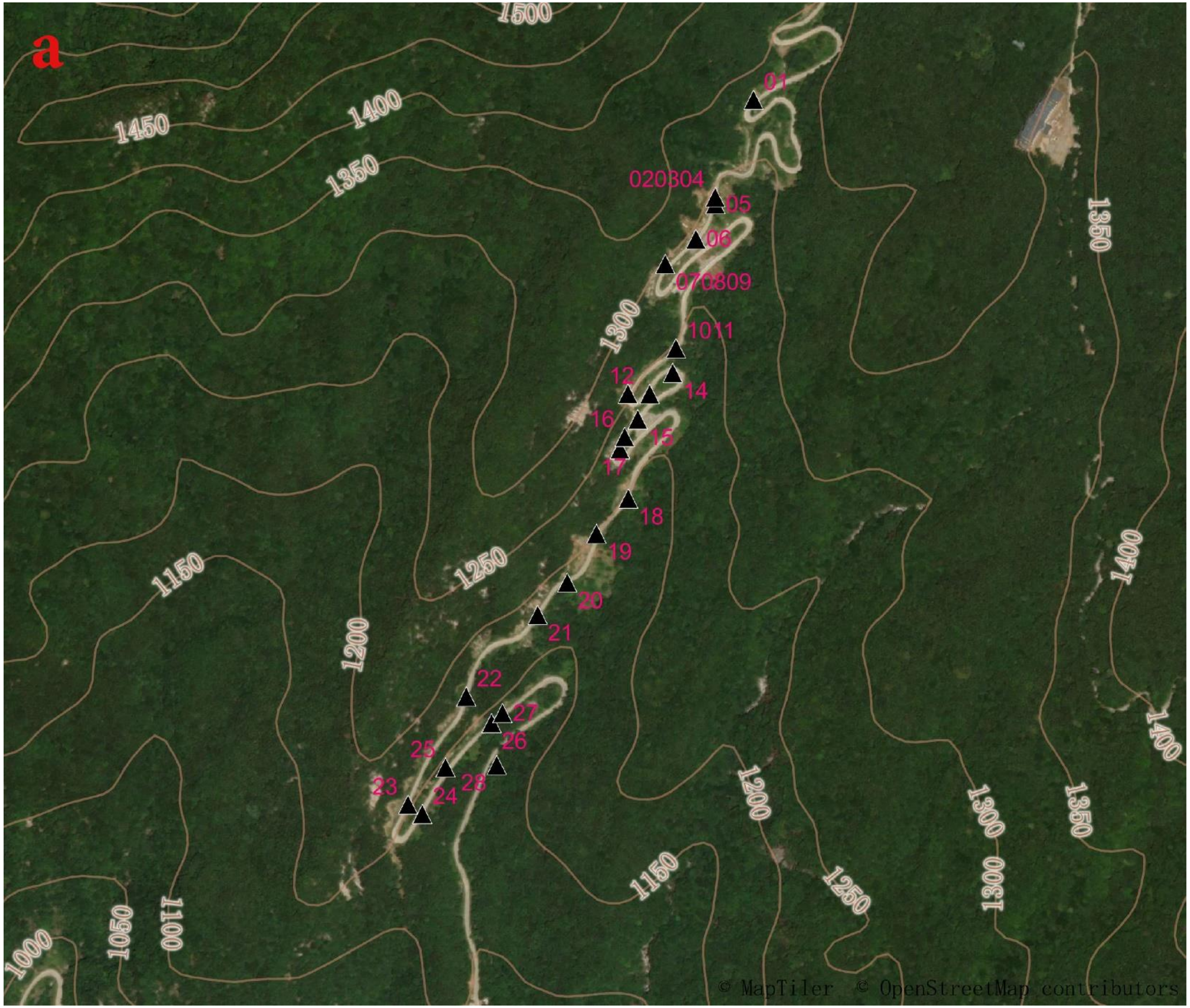

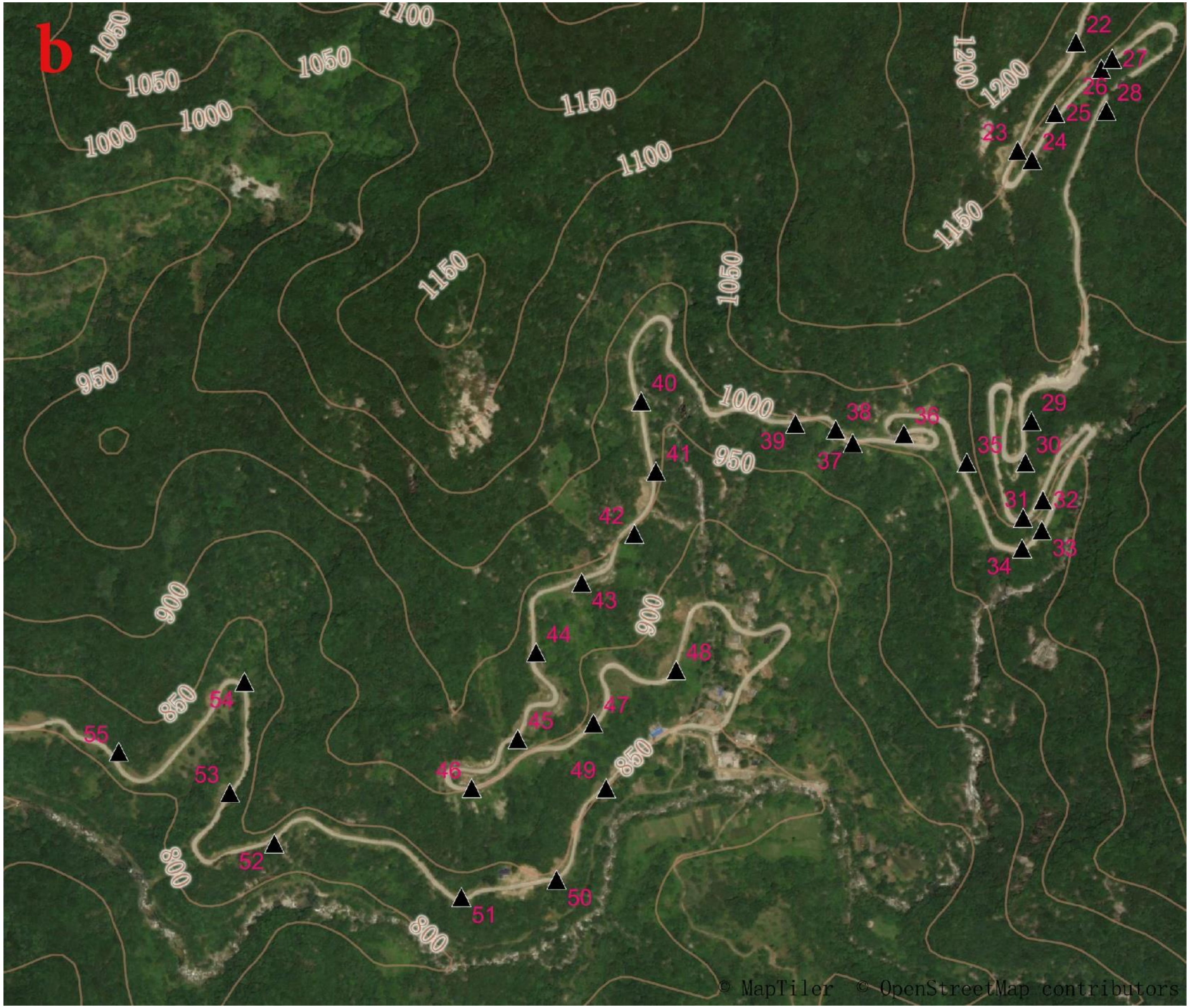

b
1050
1050
1100
1050
1150
1000
1000
1100
1200
1200
1150
1150
1050
950
1000
950
900
900
850
850
800
800
22
23
24
25
26
27
28
29
30
31
32
33
34
35
36
37
38
39
40
41
42
43
44
45
46
47
48
49
50
51
52
53
54
55
© MapTiler © OpenStreetMap contributors

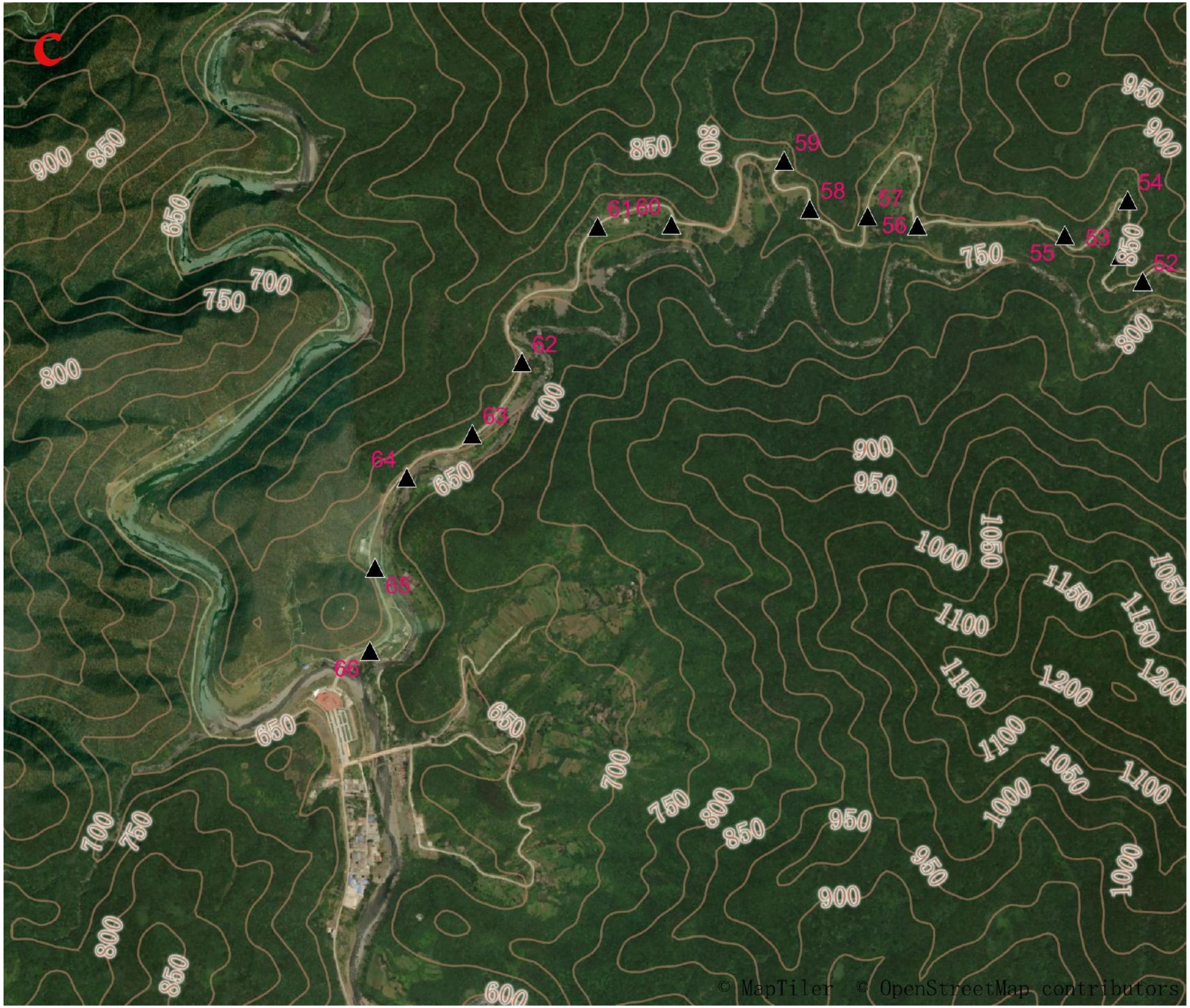

c
59
58
57
56
54
53
55
52
61
60
62
63
64
65
66
© MapTiler © OpenStreetMap contributors

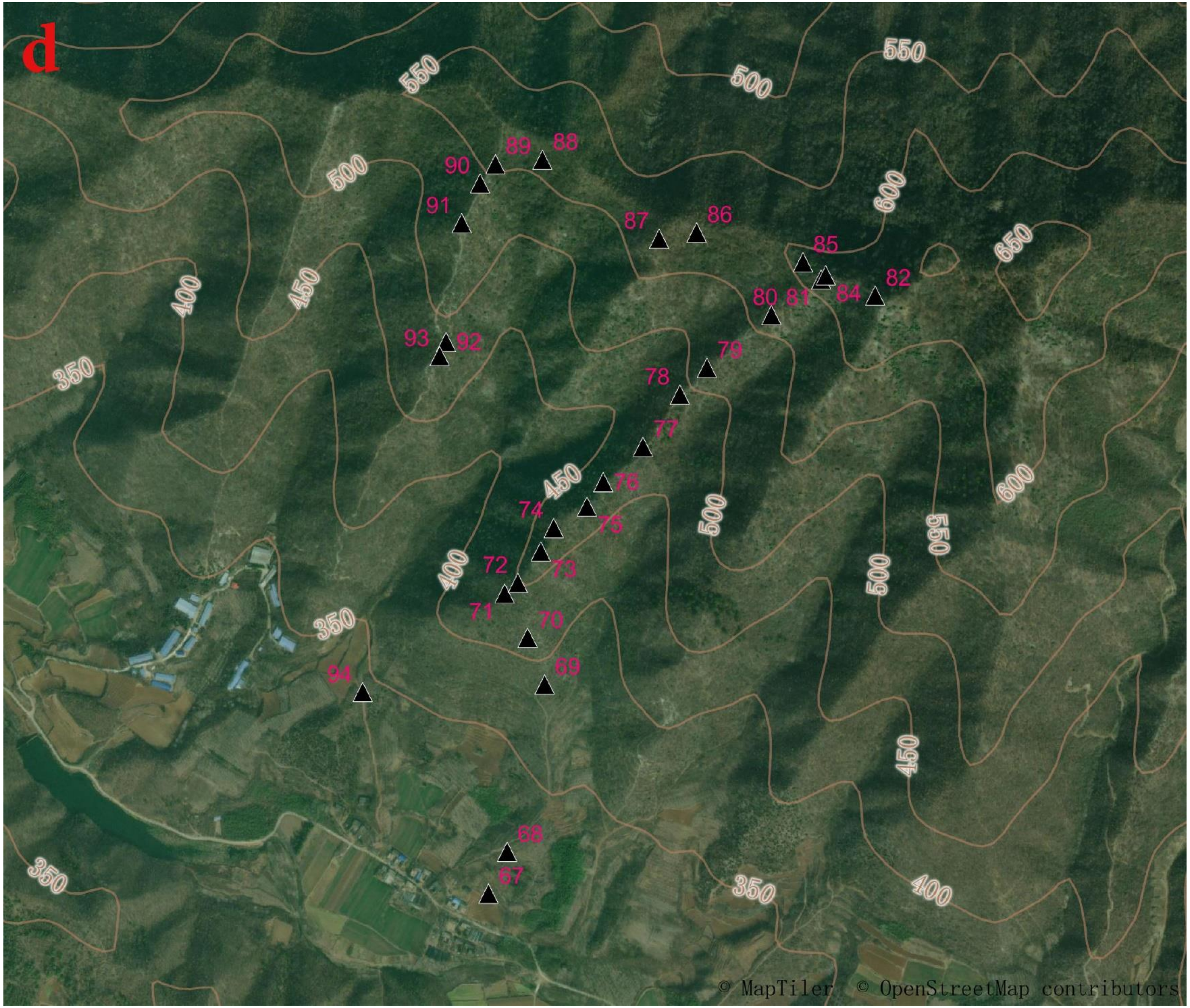
d
550
500
550
500
600
650
400
450
350
450
500
400
350
350
600
550
500
450
350
400
350
88
89
90
91
87
86
85
82
84
80
81
79
93
92
78
77
76
74
75
72
73
71
70
69
94
68
67
© MapTiler © OpenStreetMap contributors

Figure S2. Phylogenetic tree of leaf-mining insects on *Quercus variabilis* Blume

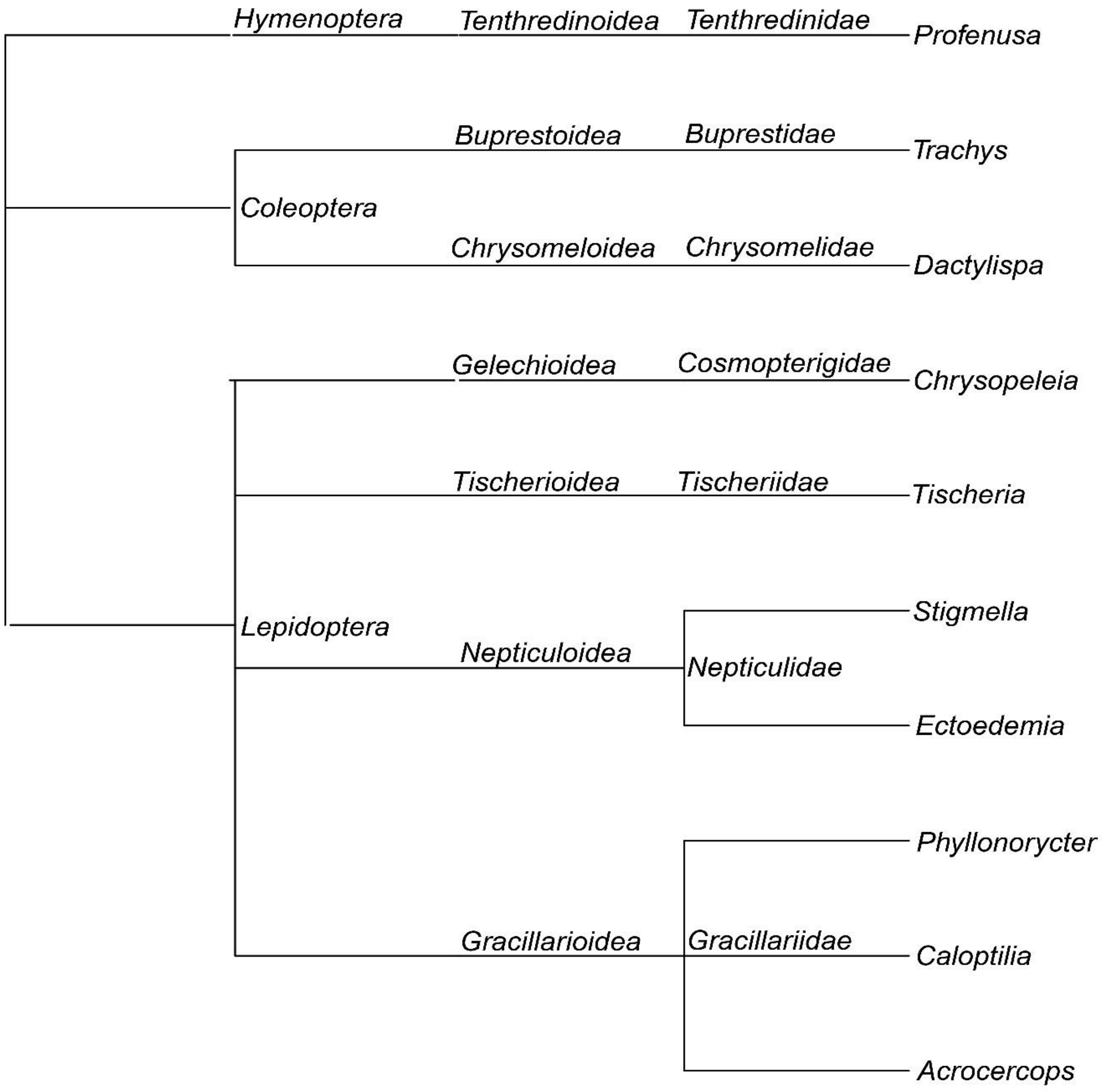

Figure S3. Clustering dendrogram of leaf-mining insects on *Quercus variabilis* Blume based on functional traits

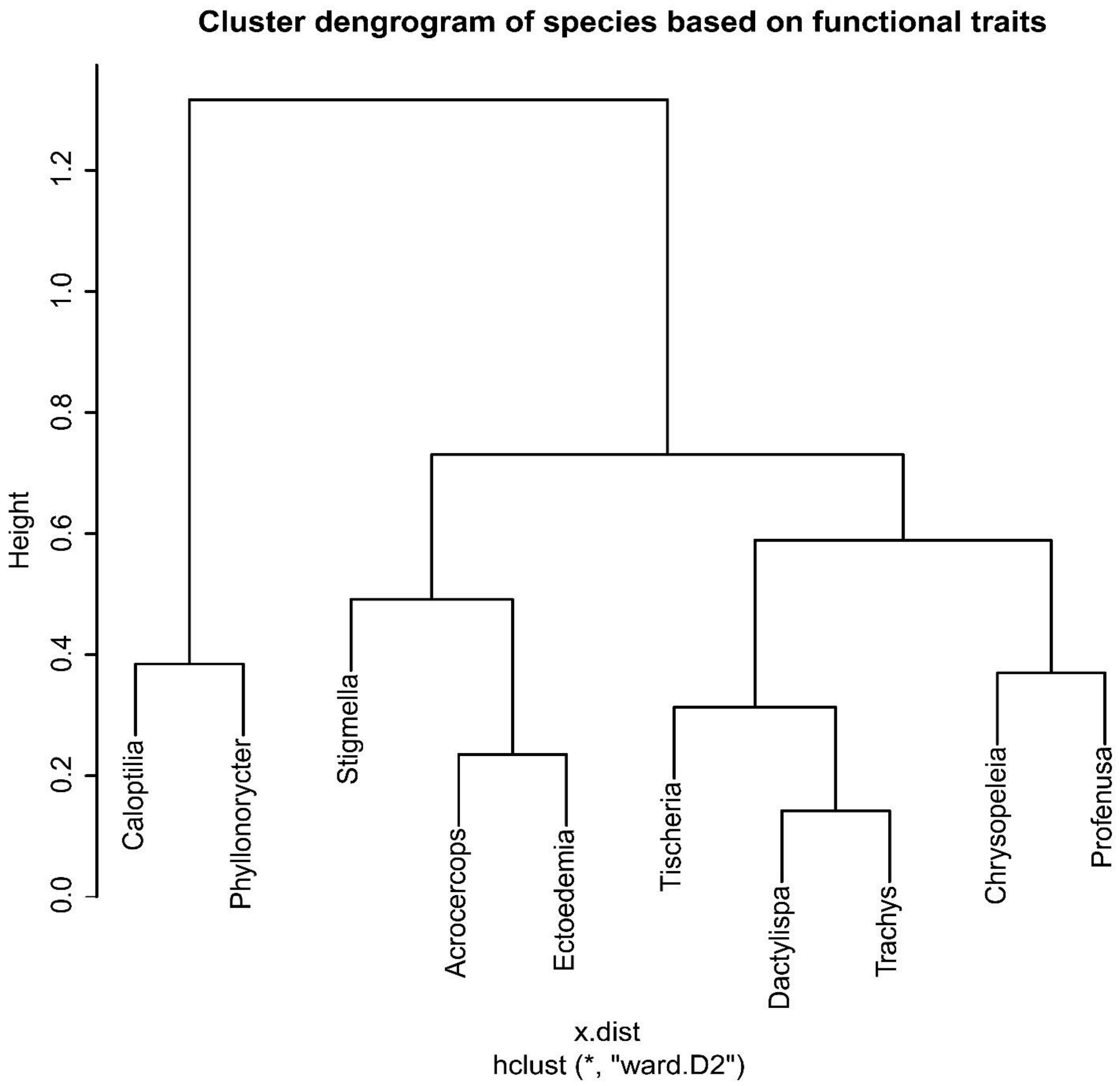

Supplement: Supplementary file 1 [file insects-14-00007-s001.zip › insects-2038290 supplementary figures.pdf]
