# Supplementary material for: Elevational Pattern of Leaf Mine Diversity on Quercus variabilis Blume at Baotianman, Henan, China
Source: Insects. 2022 Dec 21;14(1):7. doi: 10.3390/insects14010007 (PMC9861204; doi:10.3390/insects14010007)
Supplement: Supplementary file 1 [file insects-14-00007-s001.zip › insects-2038290 supplementary tables.pdf]

Table S1. Number of different mine types on *Quercus variabilis* Blume at different elevations

Sampled tree No.	Longitude (°)	Latitude (°)	Elevation (m)	LM01	LM03	LM05	LM06	LM08	LM10	LM11	LM12	LM15	LM19	Total mine number
01	111.9308709	33.511	1345	0	0	0	0	5	2	0	0	1	2	10
~~02~~	111.9302263	33.509746	1306	0	0	0	0	6	0	4	0	0	0	10
~~03~~	111.9302263	33.509746	1306	0	0	0	0	10	0	14	0	0	1	25
04	111.9302263	33.509746	1306	4	0	0	0	6	0	7	0	3	2	22
05	111.9302263	33.509657	1303	0	0	0	0	17	2	2	0	1	0	22
~~07~~	111.9294046	33.508891	1292	0	0	0	0	12	7	2	0	8	1	30
08	111.9294046	33.508891	1292	0	1	0	2	29	6	3	0	1	7	49
~~09~~	111.9294046	33.508891	1292	0	0	0	0	18	9	2	0	0	11	40
06	111.9299041	33.509209	1290	0	0	0	2	28	3	7	0	2	12	54
12	111.9287655	33.507217	1255	0	0	0	0	26	2	0	0	0	1	29
10	111.9295495	33.507795	1254	0	0	0	0	46	10	5	0	1	2	64
~~11~~	111.9295495	33.507795	1254	0	0	0	0	12	2	4	0	0	1	19
13	111.9291145	33.507208	1249	0	0	0	0	8	5	2	0	0	9	24
14	111.9294904	33.507477	1246	0	0	0	0	35	2	0	0	6	14	57
15	111.9289158	33.506881	1241	0	0	0	0	25	3	8	0	8	5	49
16	111.928701	33.506657	1236	0	0	0	2	38	5	4	0	1	7	57
17	111.9286204	33.506501	1230	2	0	0	0	57	15	10	0	5	19	108
18	111.9287385	33.505856	1208	0	0	0	2	15	7	1	0	4	10	39
19	111.9282176	33.505408	1198	0	0	0	2	31	6	2	0	0	3	44
20	111.9277343	33.504781	1194	2	0	0	0	13	6	0	0	4	2	27
21	111.927251	33.504369	1190	0	0	0	0	47	19	2	0	2	5	75
22	111.9260805	33.503326	1170	0	0	0	1	34	9	2	0	5	2	53
23	111.925114	33.501937	1160	0	0	0	0	29	3	0	0	0	2	34
25	111.925726	33.502412	1153	0	0	0	0	4	4	0	0	0	3	11
26	111.9264777	33.502977	1153	5	0	0	2	22	7	0	0	6	10	52
27	111.9266549	33.503102	1152	0	0	0	3	51	10	7	0	1	4	76
24	111.9253394	33.501812	1147	0	0	0	0	13	1	2	0	1	0	17
28	111.9265475	33.502431	1144	0	0	0	0	15	2	0	0	0	1	18
29	111.9252478	33.498425	1091	0	0	0	0	14	5	1	0	0	9	29
30	111.9251404	33.497887	1071	0	0	0	1	3	1	0	0	1	5	11
32	111.9254088	33.497395	1066	1	0	0	0	31	10	1	0	1	14	58

31	111.9250867	33.497171	1064	0	0	0	0	17	10	1	0	0	3	31
33	111.9253819	33.497006	1060	0	0	0	0	4	1	0	0	0	0	5
34	111.9250598	33.496773	1055	0	0	0	0	6	3	0	0	0	0	9
35	111.9242009	33.4979	1040	1	0	0	0	2	3	0	0	0	7	13
36	111.9232025	33.498289	1018	0	0	0	0	12	6	3	0	0	1	22
39	111.9214687	33.498449	1013	0	0	0	0	12	3	0	0	0	10	25
38	111.9221128	33.49836	1011	0	0	0	2	18	5	3	0	0	1	29
37	111.9223812	33.498181	1003	0	0	0	0	5	2	0	0	0	4	11
40	111.9190052	33.498774	970	0	0	0	0	5	9	2	0	0	7	23
41	111.9192198	33.497861	957	0	0	0	0	10	4	0	0	0	2	16
42	111.9188547	33.497055	955	0	0	0	13	31	12	13	0	2	4	75
43	111.917996	33.496437	947	0	0	0	0	17	18	1	1	6	16	59
44	111.9172446	33.495542	916	0	0	0	0	31	12	3	0	10	11	67
45	111.9169225	33.494423	915	0	0	0	0	6	10	7	0	3	7	33
46	111.9161712	33.493796	909	0	0	0	0	10	9	5	0	0	8	32
47	111.9181407	33.494611	889	1	0	0	0	14	12	0	0	3	9	39
48	111.9194825	33.495279	875	0	0	0	6	14	4	8	0	13	15	60
49	111.9183231	33.493761	858	0	0	0	0	13	10	11	0	4	26	64
50	111.9175019	33.492588	855	0	0	0	6	51	8	38	0	13	30	146
52	111.9129947	33.493128	845	0	0	0	0	4	4	1	0	0	10	19
53	111.9122973	33.493799	841	0	0	0	0	9	6	3	0	0	4	22
51	111.9159725	33.492386	839	0	0	0	0	7	2	5	0	0	8	22
54	111.9125657	33.495232	830	0	0	0	2	15	4	6	0	0	1	28
55	111.9105323	33.494354	822	4	0	0	0	31	9	10	0	5	26	85
59	111.901592	33.496436	807	0	0	0	0	7	1	2	0	0	6	16
58	111.9023855	33.495164	792	0	0	0	0	8	0	5	0	0	1	14
56	111.9058176	33.494681	790	0	0	0	1	2	1	1	0	0	1	6
57	111.9042302	33.494954	781	0	0	0	0	6	4	1	0	0	5	16
60	111.897946	33.494826	746	0	0	0	0	16	7	7	0	0	3	33
61	111.8955819	33.494818	725	0	0	0	0	1	0	3	0	1	2	7
62	111.8930784	33.491337	682	0	0	0	2	26	6	1	0	0	2	37
63	111.8914489	33.489494	669	0	0	0	2	26	9	8	0	0	7	52
64	111.8893265	33.488404	644	0	0	0	0	2	3	4	0	0	0	9

82	111.8937642	33.320967	637	0	0	0	0	0	5	1	0	0	0	6
83	111.8947666	33.321527	637	0	0	0	3	29	11	6	0	0	2	51
65	111.8882546	33.486077	636	1	0	0	0	16	0	9	0	0	2	28
66	111.88804	33.483928	633	0	0	0	1	20	2	6	0	0	8	37
84	111.8929817	33.321241	588	0	0	0	0	6	0	0	0	0	2	8
81	111.8929067	33.321187	586	0	0	0	0	20	6	4	0	2	3	35
85	111.8926226	33.321416	571	1	0	0	4	22	2	3	1	0	0	33
88	111.8884961	33.322814	563	0	0	0	17	38	8	6	0	0	5	74
80	111.8921027	33.320739	560	0	0	0	8	135	27	14	0	0	9	193
86	111.8909344	33.32183	553	10	0	0	0	17	0	0	0	0	1	28
89	111.8877459	33.32277	552	0	0	0	2	17	2	2	0	0	0	23
87	111.8903342	33.321758	550	0	0	0	2	18	6	2	0	1	1	30
90	111.887494	33.322523	537	0	0	0	10	40	12	3	0	0	5	70
91	111.8871886	33.322012	523	0	0	0	4	25	6	0	0	0	2	37
79	111.8910575	33.320071	497	0	0	0	9	38	13	2	0	0	2	64
78	111.890618	33.319731	477	0	0	0	2	14	11	3	0	0	5	35
92	111.8869044	33.320474	467	0	0	0	19	49	13	3	1	0	16	101
93	111.8867972	33.320294	463	0	0	0	6	16	4	1	1	0	0	28
77	111.8900124	33.319063	457	0	0	0	3	16	13	2	0	0	2	36
74	111.8885653	33.318032	439	0	0	0	3	4	4	0	0	0	0	11
75	111.8891013	33.318301	438	1	0	1	0	48	23	9	2	0	8	92
76	111.8893692	33.318615	437	0	0	0	10	48	7	0	0	0	3	68
73	111.888351	33.317732	436	0	0	0	2	6	3	0	0	0	1	12
72	111.8879758	33.317329	430	0	0	0	0	13	0	0	0	0	0	13
71	111.8877615	33.317194	423	0	0	0	3	25	6	0	0	0	5	39
70	111.8881151	33.316615	414	2	0	0	0	31	8	2	0	1	5	49
69	111.8883722	33.316005	383	1	0	0	3	23	5	1	0	0	6	39
94	111.885468	33.315954	348	0	0	0	15	60	16	3	0	0	27	121
68	111.8877236	33.313844	333	1	0	0	8	61	37	4	1	0	4	116
67	111.8874128	33.313306	330	0	0	0	0	8	6	2	0	0	6	22

Note: Sampled trees with the strikeout were not included in our analysis because of several tree samples at the same location

Table S2. Functional characteristics of different leaf mine types on *Quercus variabilis* Blume

LM	Genus	Oviposition	Exit	MineShape	MineDepth	VeinLimited	Frass	LinearPart	BlotchPart	Placement	Transition	MineArea (cm²)
LM01	*Acrocercops*	U	U	LB	E	N	FF	PL	OB	RM	N	5.213
LM03	*Caloptilia*	L	L	LB	LP	N	FG	PL	TeB	LT	MO	2.440
LM05	*Dactylispa*	U	U	B	FD	N	EF	N	OB	LM	N	3.703
LM06	*Ectoedemia*	U	U	LB	UP	N	FP	PL	OB	RM	N	0.690
LM08	*Phyllonorycter*	L	L	LB	LP	Y	FF	PL	TeB	RM	EP	0.665
LM10	*Stigmella*	U	U	L	UP	N	SFL	SpL	N	RM	N	0.803
LM11	*Tischeria*	U	U	B	UP	N	EF	N	OB	RM	N	2.151
LM12	*Trachys*	U	U	B	FD	N	FT	N	OB	LM	N	8.050
LM15	*Chrysopeleia*	U	U	B	UP	Y	FP	NO	OB	LI	N	0.366
LM19	*Profenusa*	U	U	B	FD	Y	FP	N	TrB	RM	N	0.898

Note: (1) Oviposition (or Exit): U upper, L lower; (2) MS (mine shape): LB linear-blotch, B blotch, L linear; (3) MD (mine depth): E epidermis, LP lower parenchyma, FD full depth, UP upper parenchyma; (4) Frass: FF free of frass, FG frass grain, EF ejection of frass, FP frass pile, SPL single frass line, FT frass thread; (5) LP (linear part): PL pure linear, SpL serpentine linear mine ; (6) BP (blotch part): OB ordinary blotch, TeB tentiform blotch, TrB trumpet blotch; (7) Placement: RM random, LT leaf tip, LI leaf inner area; (8) Transition: MO from mining to other feeding habits, EP from epidermis to parenchyma; (9) VL vein limited; (10) N No, Y Yes

Table S3. Taxonomic status of different leaf mine types on *Quercus variabilis* Blume

LM	Order	Superfamily	Family	Genus	Note
LM01	Lepidoptera	Gracillarioidea	Gracillariidae	*Acrocercops*	?
LM03	Lepidoptera	Gracillarioidea	Gracillariidae	*Caloptilia*	
LM05	Coleoptera	Chrysomeloidea	Chrysomelidae	*Dactylispa*	
LM06	Lepidoptera	Nepticuloidea	Nepticulidae	*Ectoedemia*	
LM08	Lepidoptera	Gracillarioidea	Gracillariidae	*Phyllonorycter*	
LM10	Lepidoptera	Nepticuloidea	Nepticulidae	*Stigmella*	
LM11	Lepidoptera	Tischerioidea	Tischeriidae	*Tischeria*	
LM12	Coleoptera	Buprestoidea	Buprestidae	*Trachys*	
LM15	Lepidoptera	Gelechioidea	Cosmopterigidae	*Chrysopeleia*	?
LM19	Hymenoptera	Tenthredinoidea	Tenthredinidae	*Profenusa*	?

Table S4. Number of leaf miner genera on *Quercus variabilis* Blume at different altitudes at Baotianman Scenic Area

Sampled tree No.	Longitude (º)	Latitude (º)	Elevation (m)	Leaf miner genera	Number of genera
01	111.9308709	33.51099997	1345	*Phyllonorycter, Stigmella, Chrysopeleia, Profenusa*	4
~~02~~	111.9302263	33.5097464	1306	*Phyllonorycter, Tischeria*	2
~~03~~	111.9302263	33.5097464	1306	*Phyllonorycter, Tischeria,Profenusa*	3
04	111.9302263	33.5097464	1306	*Acrocercops, Phyllonorycter, Tischeria, Chrysopeleia, Profenusa*	5
05	111.9302263	33.5096569	1303	*Phyllonorycter, Stigmella, Tischeria, Chrysopeleia*	4
~~07~~	111.9294046	33.5088909	1292	*Phyllonorycter, Stigmella, Tischeria, Chrysopeleia, Profenusa*	5
08	111.9294046	33.5088909	1292	*Caloptilia, Ectoedemia, Phyllonorycter, Stigmella, Tischeria, Chrysopeleia, Profenusa*	7
~~09~~	111.9294046	33.5088909	1292	*Phyllonorycter, Stigmella, Tischeria, Profenusa*	4
06	111.9299041	33.5092091	1290	*Ectoedemia, Phyllonorycter, Stigmella, Tischeria, Chrysopeleia, Profenusa*	6
12	111.9287655	33.5072167	1255	*Phyllonorycter, Stigmella, Profenusa*	3
10	111.9295495	33.50779471	1254	*Phyllonorycter, Stigmella, Tischeria, Chrysopeleia, Profenusa*	5
~~11~~	111.9295495	33.50779471	1254	*Phyllonorycter, Stigmella, Tischeria, Profenusa*	4
13	111.9291145	33.50720808	1249	*Phyllonorycter, Stigmella, Tischeria, Profenusa*	4
14	111.9294904	33.50747694	1246	*Phyllonorycter, Stigmella, Chrysopeleia, Profenusa*	4
15	111.9289158	33.50688123	1241	*Phyllonorycter, Stigmella, Tischeria, Chrysopeleia, Profenusa*	5
16	111.928701	33.50665728	1236	*Ectoedemia, Phyllonorycter, Stigmella, Tischeria, Chrysopeleia, Profenusa*	6

17	111.9286204	33.50650058	1230	*Acrocercops Phyllonorycter, Stigmella, Tischeria, Chrysopeleia, Profenusa*	6
18	111.9287385	33.5058563	1208	*Ectoedemia Phyllonorycter, Stigmella, Tischeria, Chrysopeleia, Profenusa*	6
19	111.9282176	33.50540832	1198	*Ectoedemia, Phyllonorycter, Stigmella, Tischeria, Profenusa*	5
20	111.9277343	33.50478138	1194	*Acrocercops, Phyllonorycter, Stigmella, Chrysopeleia, Profenusa*	5
21	111.927251	33.50436924	1190	*Phyllonorycter, Stigmella, Tischeria, Chrysopeleia, Profenusa*	5
22	111.9260805	33.50332555	1170	*Ectoedemia, Phyllonorycter, Stigmella, Tischeria, Chrysopeleia, Profenusa*	6
23	111.925114	33.50193748	1160	*Phyllonorycter, Stigmella, Profenusa*	3
25	111.925726	33.50241234	1153	*Phyllonorycter, Stigmella, Profenusa*	3
26	111.9264777	33.50297683	1153	*Acrocercops, Ectoedemia, Phyllonorycter, Stigmella, Chrysopeleia, Profenusa*	6
27	111.9266549	33.50310228	1152	*Ectoedemia, Phyllonorycter, Stigmella, Tischeria, Chrysopeleia, Profenusa*	6
24	111.9253394	33.50181236	1147	*Phyllonorycter, Stigmella, Tischeria, Chrysopeleia*	4
28	111.9265475	33.50243092	1144	*Phyllonorycter, Stigmella, Profenusa*	3
29	111.9252478	33.49842451	1091	*Phyllonorycter, Stigmella, Tischeria, Profenusa*	4
30	111.9251404	33.49788738	1071	*Ectoedemia, Phyllonorycter, Stigmella, Chrysopeleia, Profenusa*	5
32	111.9254088	33.4973953	1066	*Acrocercops, Phyllonorycter, Stigmella, Tischeria, Chrysopeleia, Profenusa*	6
31	111.9250867	33.49717127	1064	*Phyllonorycter, Stigmella, Tischeria, Profenusa*	4
33	111.9253819	33.49700592	1060	*Phyllonorycter, Stigmella*	2
34	111.9250598	33.49677294	1055	*Phyllonorycter, Stigmella*	2

35	111.9242009	33.49790009	1040	*Acrocercops, Phyllonorycter, Stigmella, Profenusa*	4
36	111.9232025	33.49828872	1018	*Phyllonorycter, Stigmella, Tischeria, Profenusa*	4
39	111.9214687	33.49844868	1013	*Phyllonorycter, Stigmella, Profenusa*	3
38	111.9221128	33.49835959	1011	*Ectoedemia, Phyllonorycter, Stigmella, Tischeria, Profenusa*	5
37	111.9223812	33.49818075	1003	*Phyllonorycter, Stigmella, Profenusa*	3
40	111.9190052	33.49877398	970	*Phyllonorycter, Stigmella, Tischeria, Profenusa*	4
41	111.9192198	33.49786112	957	*Phyllonorycter, Stigmella, Profenusa*	3
42	111.9188547	33.49705536	955	*Ectoedemia, Phyllonorycter, Stigmella, Tischeria, Chrysopeleia, Profenusa*	6
43	111.917996	33.49643733	947	*Phyllonorycter, Stigmella, Tischeria, Trachys, Chrysopeleia, Profenusa*	6
44	111.9172446	33.49554189	916	*Phyllonorycter, Stigmella, Tischeria, Chrysopeleia, Profenusa*	5
45	111.9169225	33.49442287	915	*Phyllonorycter, Stigmella, Tischeria, Chrysopeleia, Profenusa*	5
46	111.9161712	33.49379598	909	*Phyllonorycter, Stigmella, Tischeria, Profenusa*	4
47	111.9181407	33.49461139	889	*Acrocercops, Phyllonorycter, Stigmella, Chrysopeleia, Profenusa*	5
48	111.9194825	33.49527892	875	*Ectoedemia, Phyllonorycter, Stigmella, Tischeria, Chrysopeleia, Profenusa*	6
49	111.9183231	33.49376111	858	*Phyllonorycter, Stigmella, Tischeria, Chrysopeleia, Profenusa*	5
50	111.9175019	33.49258811	855	*Ectoedemia, Phyllonorycter, Stigmella, Tischeria, Chrysopeleia, Profenusa*	6
52	111.9129947	33.49312811	845	*Phyllonorycter, Stigmella, Tischeria, Profenusa*	4
53	111.9122973	33.4937993	841	*Phyllonorycter, Stigmella, Tischeria, Profenusa*	4

51	111.9159725	33.49238607	839	*Phyllonorycter, Stigmella, Tischeria, Profenusa*	4
54	111.9125657	33.49523154	830	*Ectoedemia, Phyllonorycter, Stigmella, Tischeria, Profenusa*	5
55	111.9105323	33.49435397	822	*Acrocercops, Phyllonorycter, Stigmella, Tischeria, Chrysopeleia, Profenusa*	6
59	111.901592	33.49643564	807	*Phyllonorycter, Stigmella, Tischeria, Profenusa*	4
58	111.9023855	33.49516441	792	*Phyllonorycter, Tischeria, Profenusa*	3
56	111.9058176	33.49468054	790	*Ectoedemia, Phyllonorycter, Stigmella, Tischeria, Profenusa*	5
57	111.9042302	33.49495372	781	*Phyllonorycter, Stigmella, Tischeria, Profenusa*	4
60	111.897946	33.49482566	746	*Phyllonorycter, Stigmella, Tischeria, Profenusa*	4
61	111.8955819	33.49481779	725	*Phyllonorycter, Tischeria, Chrysopeleia, Profenusa*	4
62	111.8930784	33.49133711	682	*Ectoedemia, Phyllonorycter, Stigmella, Tischeria, Profenusa*	5
63	111.8914489	33.48949412	669	*Ectoedemia, Phyllonorycter, Stigmella, Tischeria, Profenusa*	5
64	111.8893265	33.48840351	644	*Phyllonorycter, Stigmella, Tischeria*	3
65	111.8882546	33.48607682	636	*Acrocercops, Phyllonorycter, Tischeria, Profenusa*	4
66	111.88804	33.48392846	633	*Ectoedemia, Phyllonorycter, Stigmella, Tischeria, Profenusa*	5

Note: Sampled trees with the strikeout were not included in our analysis because of several tree samples at the same location.

Table S5. Number of leaf miner genera on *Quercus variabilis* Blume at different altitudes at Houyemiao, Qiliping County

Sampled tree No.	Longitude (º)	Latitude (º)	Elevation (m)	Leaf miner genera	Number of genera
82	111.8937642	33.32096679	637	*Stigmella, Tischeria*	2
83	111.8947666	33.32152688	637	*Ectoedemia, Phyllonorycter, Stigmella, Tischeria, Profenusa*	5
84	111.8929817	33.32124085	586	*Phyllonorycter, Stigmella, Tischeria, Chrysopeleia, Profenusa*	2
81	111.8929067	33.32118707	588	*Phyllonorycter, Profenusa*	5
85	111.8926226	33.32141599	571	*Acrocercops, Ectoedemia, Phyllonorycter, Stigmella, Tischeria, Trachys*	6
88	111.8884961	33.32281375	563	*Ectoedemia, Phyllonorycter, Stigmella, Tischeria, Profenusa*	5
80	111.8921027	33.32073906	560	*Ectoedemia, Phyllonorycter, Stigmella, Tischeria, Profenusa*	5
86	111.8909344	33.32182973	553	*Acrocercops, Phyllonorycter, Profenusa*	3
89	111.8877459	33.3227695	552	*Ectoedemia, Phyllonorycter, Stigmella, Tischeria,*	4
87	111.8903342	33.32175839	550	*Ectoedemia, Phyllonorycter, Stigmella, Tischeria, Chrysopeleia, Profenusa*	6
90	111.887494	33.32252302	537	*Ectoedemia, Phyllonorycter, Stigmella, Tischeria, Profenusa*	5
91	111.8871886	33.32201197	523	*Ectoedemia, Phyllonorycter, Stigmella, Profenusa*	4
79	111.8910575	33.32007146	497	*Ectoedemia, Phyllonorycter, Stigmella, Tischeria, Profenusa*	5
78	111.890618	33.31973089	477	*Ectoedemia, Phyllonorycter, Stigmella, Tischeria, Profenusa*	5
92	111.8869044	33.3204738	467	*Ectoedemia, Phyllonorycter, Stigmella, Tischeria, Trachys, Profenusa*	6
93	111.8867972	33.32029449	463	*Ectoedemia, Phyllonorycter, Stigmella, Tischeria, Trachys,*	5
77	111.8900124	33.31906301	457	*Ectoedemia, Phyllonorycter, Stigmella, Tischeria, Profenusa*	5
74	111.8885653	33.31803248	439	*Ectoedemia, Phyllonorycter, Stigmella*	3
75	111.8891013	33.31830119	438	*Acrocercops, Dactylispa, Phyllonorycter, Stigmella, Tischeria, Tischeria, Profenusa*	7
76	111.8893692	33.31861496	437	*Ectoedemia, Phyllonorycter, Stigmella, Profenusa*	4
73	111.888351	33.31773213	436	*Ectoedemia, Phyllonorycter, Stigmella, Profenusa*	4
72	111.8879758	33.31732875	430	*Phyllonorycter*	1
71	111.8877615	33.31719436	423	*Ectoedemia, Phyllonorycter, Stigmella, Profenusa*	4

70	111.8881151	33.31661546	414	*Acrocercops, Phyllonorycter, Stigmella, Tischeria, Chrysopeleia, Profenusa*	6
69	111.8883722	33.31600524	383	*Acrocercops, Ectoedemia, Phyllonorycter, Stigmella, Tischeria, Profenusa*	6
94	111.885468	33.31595385	348	*Ectoedemia, Phyllonorycter, Stigmella, Tischeria, Profenusa*	5
68	111.8877236	33.31384375	333	*Acrocercops, Ectoedemia, Phyllonorycter, Stigmella, Tischeria, Trachys, Profenusa*	7
67	111.8874128	33.31330573	330	*Phyllonorycter, Stigmella, Tischeria, Profenusa*	4

Table S6. Moran's index output from the 'modifiedttest' function in the "SpatialPack" R package

Number of cells for Moran's index	Moran Index									
	Elevation	Taxonomic Hill numbers			Phylogenetic Hill numbers			Functional Hill numbers		
		q=0	q=1	q=2	q=0	q=1	q=2	q=0	q=1	q=2
1	1.137	0.004	0.102	0.173	0.029	0.190	0.226	0.010	0.058	0.093
2	0.762	-0.082	-0.021	0.034	-0.087	0.058	0.123	-0.096	-0.110	-0.100
3	0.322	-0.032	-0.155	-0.224	-0.034	-0.175	-0.194	-0.016	-0.021	-0.056
4	0.168	0.045	0.051	-0.022	0.123	0.066	-0.055	0.057	0.190	0.249
5	0.061	-0.118	-0.167	-0.132	-0.074	-0.115	-0.095	-0.132	-0.207	-0.227
6	-0.016	0.101	-0.193	-0.291	0.056	-0.177	-0.203	0.132	-0.001	-0.099
7	-0.080	0.027	-0.175	-0.304	-0.058	-0.303	-0.375	0.016	-0.037	-0.056
8	-0.157	-0.014	-0.146	-0.258	-0.046	-0.335	-0.420	-0.037	-0.095	-0.152
9	-0.315	0.057	-0.074	-0.185	-0.018	-0.332	-0.394	0.054	-0.039	-0.106
10	-0.675	-0.082	0.100	0.125	-0.005	0.109	0.075	-0.075	0.012	0.050
11	-1.081	0.010	0.057	0.090	0.033	0.097	0.113	-0.002	0.049	0.061
12	-1.571	-0.008	-0.006	0.053	-0.090	-0.003	0.070	0.003	-0.086	-0.089
13	-1.981	0.032	-0.044	-0.071	0.028	-0.054	-0.036	0.025	-0.025	-0.059
